# Rosemary (*Rosmarinus officinalis* L.) Glycolic Extract Protects Liver Mitochondria from Oxidative Damage and Prevents Acetaminophen-Induced Hepatotoxicity

**DOI:** 10.3390/antiox12030628

**Published:** 2023-03-03

**Authors:** Natalia S. S. Guimarães, Vyctória S. Ramos, Laura F. L. Prado-Souza, Rayssa M. Lopes, Gabriel S. Arini, Luís G. P. Feitosa, Ricardo R. Silva, Iseli L. Nantes, Debora C. Damasceno, Norberto P. Lopes, Tiago Rodrigues

**Affiliations:** 1 Interdisciplinary Center of Biochemistry Investigation, University of Mogi das Cruzes (UMC), Mogi das Cruzes CEP 08780-911, SP, Brazil; 2 Center for Natural and Human Sciences, Federal University of ABC, Santo André CEP 09210-580, SP, Brazil; 3 NPPNS, Department of Biomolecular Sciences, Faculty of Pharmaceutical Sciences of Ribeirão Preto, University of São Paulo, Ribeirão Preto CEP 14040-900, SP, Brazil; 4 Laboratory of Experimental Research on Gynecology and Obstetrics, Sao Paulo State University (UNESP), Botucatu CEP 18618-687, SP, Brazil

**Keywords:** antioxidant, acetaminophen, hepatoprotection, mitochondria, oxidative stress, rosemary

## Abstract

*Rosmarinus officinalis* L. (rosemary) is an aromatic culinary herb. Native to the Mediterranean region, it is currently cultivated worldwide. In addition to its use as a condiment in food preparation and in teas, rosemary has been widely employed in folk medicine and cosmetics. Several beneficial effects have been described for rosemary, including antimicrobial and antioxidant activities. Here, we investigated the mechanisms accounting for the antioxidant activity of the glycolic extract of *R. officinalis* (*Ro*) in isolated rat liver mitochondria (RLM) under oxidative stress conditions. We also investigated its protective effect against acetaminophen-induced hepatotoxicity in vivo. A crude extract was obtained by fractionated percolation, using propylene glycol as a solvent due to its polarity and cosmeceutical compatibility. The quantification of substances with recognized antioxidant action revealed the presence of phenols and flavonoids. Dereplication studies carried out through LC-MS/MS and GC-MS, supported by The Global Natural Product Social Molecular Networking (GNPS) platform, annotated several phenolic compounds, confirming the previous observation. In accordance, *Ro* decreased the production of reactive oxygen species (ROS) elicited by Fe^2+^ or *t*-BOOH and inhibited the lipid peroxidation of mitochondrial membranes in a concentration-dependent manner in RLM. Such an effect was also observed in liposomes as membrane models. *Ro* also prevented the oxidation of mitochondrial protein thiol groups and reduced glutathione (GSH). In model systems, *Ro* exhibited a potent scavenger activity toward 2,2′-diphenyl-1-picrylhydrazyl (DPPH) radicals and superoxide anions. It also demonstrated an Fe^2+^ chelating activity. Moreover, *Ro* did not exhibit cytotoxicity or dissipate the mitochondrial membrane potential (∆Ψ) in rat liver fibroblasts (BRL3A cells). To evaluate whether such antioxidant protective activity observed in vitro could also be achieved in vivo, a well-established model of hepatotoxicity induced by acute exposure to acetaminophen (AAP) was used. This model depletes GSH and promotes oxidative-stress-mediated tissue damage. The treatment of rats with 0.05% *Ro*, administered intraperitoneally for four days, resulted in inhibition of AAP-induced lipid peroxidation of the liver and the prevention of hepatotoxicity, maintaining alanine and aspartate aminotransferase (ALT/AST) levels equal to those of the normal, non-treated rats. Together, these findings highlight the potent antioxidant activity of rosemary, which is able to protect mitochondria from oxidative damage in vitro, and effects such as the antioxidant and hepatoprotective effects observed in vivo.

## 1. Introduction

The Lamiaceae family contains a set of plants, often cultivated as herbs, whose leaves can be used as seasonings and whose essential oils are used as flavors in cosmeceuticals [1]. Among these plants, *Rosmarinus officinalis* L. stands out. Popularly known as rosemary (or alecrim in Brazil), *R. officinalis* is native to the Mediterranean region and is currently found worldwide. It has been extensively studied for its use in food and as a spice and/or preservative, in which it operates by inhibiting microbial growth and oxidative reactions [2]. The study of medicinal plants and herbs has proved to be an efficient strategy for prospecting novel compounds with therapeutic potential [3,4]. In this regard, a large number of secondary metabolites have been isolated and identified from *Rosmarinus* spp., including essential oils, flavonoids, tannins, terpenes, and phenolic acids [5,6,7]. In addition, carnosol, carnosic acid, and rosmarinic acids, which are reported to be the main components of rosemary, account for many of its biological activities [8,9,10]. The production of these bioactive secondary metabolites by rosemary depends on several factors, such as the plant part, climatic conditions and soil nutrients, humidity, temperature, water availability, and others [11]. Due to the large number of chemical studies carried out on *R. officinalis*, we opted for a dereplication strategy to avoid re-isolations [12]. Hyphenated liquid chromatography with electrospray ionization mass spectrometry (LC-ESI-MS/MS) is currently the most suitable technique for metabolomics approaches [13]. However, when analyzing extracts with an antioxidative potential, it is important to follow the oxidation processes at the source of the ESI in detail since substances with a low oxidation potential can lose electrons, changing the expected mass balance and leading to erroneous interpretations of chemical structures [14,15].

Furthermore, *R. officinalis* has long been used as a medicinal plant. In this context, a plethora of biological effects and health benefits have been attributed to rosemary essential oil, leaf extracts, and isolated substances, including antibacterial [16,17,18], antifungal [19,20], anti-inflammatory [21,22], antiatherogenic [23,24], antiangiogenic [25,26], antihypertensive [27], antiulcer [28], anti-diabetic [29,30], anticancer [31], and other effects (reviewed elsewhere [32,33]). In addition to these effects, this plant is also well known for its powerful antioxidant activity (reviewed in [34]). This is expected, as phytochemical analyses of rosemary extracts and essential oils have revealed several substances with recognized antioxidant properties, such as flavonoids and phenolic compounds [35,36]. In fact, such antioxidant activities of *R. officinalis* might help to explain some of the biological effects described above. The pathophysiology of several human diseases is related to the overproduction of reactive oxygen species (ROS) or a decreased capacity of the antioxidant defense system, resulting in oxidative stress, cellular/tissue damage, and, ultimately, organ dysfunction [37]. In this regard, it has been shown that *R. officinalis* exhibits hepatoprotective effects in different models of xenobiotic-induced liver toxicity, including carbon tetrachloride (CCl_4_) [38,39,40,41], creosote [38], azathioprine [39], hexavalent chromium [40], streptozotocin [41], and cyclophosphamide [42]. However, its protective effect against acetaminophen-induced hepatotoxicity has not been clearly demonstrated yet. Acetaminophen (AAP) is an anti-inflammatory and anti-pyretic drug, and AAP overdose is associated with acute liver failure [43]. During cytochrome P_450_ liver metabolism, AAP is transformed in the highly reactive metabolite N-acetyl-p-benzoquinone imine (NAPQI), which oxidizes GSH [44] and causes oxidative stress.

Although the antioxidant activity of *R. officinalis* has been extensively studied in several biological systems and toxicity models, its potential protective effect on mitochondria under oxidative stress conditions has not been investigated. Despite the central role they play in cellular metabolism and energy production, mitochondria are also often associated with oxidative status since superoxide radical anions (O_2_^•−^) are continuously generated during electron transport in the respiratory chain. This radical is counteracted by the antioxidant defense system, with the reducing power provided by reduced glutathione (GSH) and NAD(P)H [45]. However, excessive O_2_^•−^ formation and, consequently, its conversion to hydrogen peroxide (H_2_O_2_), can produce the extremely reactive hydroxyl radical (^•^OH) by means of the Fenton reactions, which are catalyzed by Fe^2+^ and other transition metals [46]. Thus, the investigation of the effects of *R. officinalis* in such a complex mitochondrial scenario can further elucidate the molecular mechanisms of antioxidant protection in biological systems. Here, we investigated the antioxidant properties of *R. officinalis* leaf extracts in rat liver mitochondria and addressed its protective effect against AAP-induced hepatotoxicity in rats.

## 2. Materials and Methods

### 2.1. Chemicals, Plant Source, and Extract Preparation

All reagents used in this study were of the highest commercially available grade of purity. Aqueous solutions were prepared using type I water obtained using a Milli-Q system (Millipore, USA). *Rosmarinus officinalis* L. species were collected in Brazil (GPS localization: 760 m, 23°29840′ S) during the winter (August) of 2018. After botanical identification, voucher specimens were deposited at the Herbarium Mogiense (University of Mogi das Cruzes, Brazil). After drying at 40 °C in a ventilated drying oven, 100 g of selected leaves were submitted to fractionated percolation using a mixture of propylene glycol/water (7/3) as solvent, resulting in 100 mL of *Rosmarinus officinalis* glycolic extract (*Ro*). This extract was considered a 100% crude extract for subsequent concentration calculations.

### 2.2. Isolation of Mitochondria, Animal Treatments, and Sample Preparation

Liver mitochondria were isolated from rats by differential centrifugation in isosmotic media, as previously described [47]. Adult male Wistar rats weighing approximately 180 g were randomly divided into four groups (n = 7), kept in a 12/12 h light/dark cycle, and fed ad libitum. All experiments involving animals were conducted in accordance with the guide for the care and use of laboratory animals (NIH Guidelines) and were previously approved by the Animal Ethics Committee of University of Mogi das Cruzes (CEUA). In order to induce hepatotoxicity, the AAP group received a single dose of 900 mg/kg of acetaminophen (Sigma-Aldrich, St. Louis, MI, USA) intraperitoneally. Animals were sacrificed 4 h after drug administration. The control group (C) received the same volume of solvent used for AAP dissolution, i.e., dimethyl sulfoxide (DMSO; Sigma-Aldrich, USA). The other two groups received 0.1 mL of 0.5% *Ro* intraperitoneally once per day in the morning for four consecutive days. One of these groups (*Ro*/AAP) also received AAP on the last day, as described above, 4 h before sacrifice. The animals were anesthetized with xylazine (5 mg/kg) and ketamine (60 mg/kg) for cardiac blood collection. The blood was immediately centrifuged at 700× *g* for 5 min to achieve serum separation. For homogenate preparation, the livers were removed and cut into small fragments in a buffer containing 250 mmol/L sucrose, 1 mmol/L EGTA, and HEPES- KOH 10 mmol/L at a pH of 7.4 at 4 °C. After washing the samples twice with the same medium, the tissues were homogenized using a Potter-Elvehjem. This was followed by centrifugation at 580× *g* for 5 min at 4 °C. The supernatant, called a homogenate, was immediately frozen at −70 °C for subsequent experiments.

### 2.3. Preparation of PCPECL Liposomes

The phospholipids phosphatidylcholine, phosphatidylethanolamine, and cardiolipin (PCPECL, 5:3:2 ratio) were dissolved in CHCl_3_, which was further evaporated under argon flux. The lipidic film was hydrated with 10 mM of cold sodium phosphate buffer at a pH of 7.4 and vortexed. To obtain the liposome solution (1 mM), lipids were submitted to sonication for 30 min at 4 °C using a Ney Ultrasonik (J. M. Ney Co., Bloomfield, CT, USA).

### 2.4. Total Phenols and Flavonoids

The soluble phenol derivatives were estimated using the Folin–Ciocalteu method [48] and expressed as μM of gallic acid equivalents based on a standard curve [49]. For flavonoid quantification, an aliquot was incubated in a medium containing 60 μL of glacial acetic acid, 1.0 mL of pyridine H_2_O:AlCl_3_ 12% solution (17:80:3), and 1.24 mL of DMSO:H_2_O (1:1) for 5 min at 25 °C. The absorbance of the chromophore produced in this reaction was determined spectrophotometrically at 420 nm, and the flavonoid content was determined based on a standard curve and expressed as µM of quercetin equivalents.

### 2.5. DPPH Assay, Fe^2+^, and Superoxide Scavenger Activity

The reduction of the 1,1′-diphenyl-2-picrylhydrazyl radical (DPPH, Sigma-Aldrich, USA) by *Ro* was accompanied by photometric measurement in a UV1800 spectrophotometer (Shimadzu, Kyoto, Japan). An amount of 1.5 mL of 40 mM sodium acetate pH 5.5 was mixed into the reaction with 1.0 mL of absolute ethanol containing DPPH to achieve a final concentration of 0.1 mM. After incubation with different *Ro* concentrations for 5 min at 25 °C, the final absorbance was measured at 517 nm. Quercetin (Q, Sigma-Aldrich, USA) was used as reference. The amount of Fe^2+^ was quantified photometrically at 535 nm using 0.2 mM bathophenanthroline disulfonic acid (Sigma-Aldrich, USA) [50] in a competitive assay with *Ro*. At last, the xanthine/xanthine oxidase system was used to generate O_2_^•−^. The scavenger activity of *Ro* was estimated by the inhibition of the nitroblue tetrazolium (NBT, Sigma-Aldrich, USA) reduction. After adding 0.08 U/mL xanthine oxidase to a phosphate buffer at a pH of 7.5, which contained 0.05 mM EDTA, 0.2 mM hypoxanthine, and 0.1 mM NBT, the absorbance was measured at 540 nm after 20 min of incubation at 37 °C (Shimadzu UV1800 Spectrophotometer, Tokyo, Japan).

### 2.6. Reactive Oxygen Species (ROS)

The production of mitochondrial ROS was estimated kinetically using 2′,7′-dichlorodihydrofluorescein diacetate (H_2_DCFDA, Sigma-Aldrich, USA) [51], as previously described [52]. Briefly, mitochondria (1 mg/mL) were incubated in a buffer containing 125 mM sucrose, 65 mM KCl, and 10 mM HEPES-KOH at a pH of 7.4, plus 1 µM H_2_DCFDA at 30 °C, under continuous stirring in the presence of 5 mM potassium succinate (Sigma-Aldrich, USA) (plus 2 μM rotenone, Sigma-Aldrich, USA). The fluorescence emissions were recorded in a Hitachi F-2500 Spectrophotometer (Tokyo, Japan) at 503/529 nm for excitation/emission wavelength pairs, respectively.

### 2.7. Lipid Oxidation

The liposomes (1 mM), mitochondria (1 mg/mL), or liver homogenates (1 mg/mL) were incubated with or without different *Ro* concentrations at 37 °C for 30 min, and with 50 μM Fe(NH_4_)_2_(SO)_4_ (plus 2.0 mM sodium citrate) or 0.6 mM *t*-BOOH as inducers of oxidative stress. Following incubation, for a thiobarbituric acid reactive substances (TBARS) assay, 1% thiobarbituric acid (TBA, Sigma-Aldrich, USA) was prepared by adding 50 mM NaOH, 15 μL of 10 M NaOH, and 75 μL of 20% H_3_PO_4_ to each sample, followed by further incubation for 20 min at 85 °C. The MDA-TBA complex was extracted with 2 mL of *n*-butanol, and the absorbance was measured at 535 nm. The TBARS were calculated from ε = 1.56 × 10^5^ M^−1^.cm^−1^, as described in [53]. Lipid hydroperoxides (LOOH) were also quantified in isolated mitochondria using xylenol orange, as previously described in [54]. When applied, the percentages of inhibition by *Ro* were calculated in relation to positive controls (*t*-BOOH or Fe^2+^) which were considered to achieve 100% inhibition.

### 2.8. GSH and Protein Thiol Groups

After 30 min of incubation at 37 °C in a medium containing 125 mM sucrose, 65 mM KCl, and 10 mM HEPES-KOH at a pH of 7.4, plus 5.0 mM potassium succinate and 2.0 μM rotenone, the mitochondrial suspensions or liver homogenates were treated with 0.5 mL of 13% trichloroacetic acid and centrifuged at 400× *g* for 5 min. For the GSH assay, aliquots (100 µL) of the supernatant were mixed with 1.8 mL of a 0.1 M sodium phosphate buffer at a pH of 8.0 containing 5 mM EGTA plus 100 μL of 1 mg/mL o-phthalaldehyde (Sigma-Aldrich, USA). After 15 min of incubation, the fluorescence emissions were detected at 350/420 nm (excitation/emission) in a Hitachi F-2500 (Japan) fluorescence spectrophotometer [55]. The reduced thiol groups of proteins were quantified using 5,5′-dithiobis(2-nitrobenzoic acid) (DTNB, Sigma-Aldrich, USA) [56]. The pellet obtained from the acid precipitation described above was suspended with 1 mL of a 0.5 M potassium phosphate buffer at a pH of 7.6 which contained 0.7% SDS. After adding 100 μM DTNB, the absorbance was measured at 412 nm and the amount of the reduced thiol groups was calculated from ε = 13,600 M^−1^.cm^−1^ [57].

### 2.9. Cell Culture and Cellular Assays

BRL 3A cells (CRL-1442, ATCC) were cultivated in high-glucose Dulbecco’s modified Eagle’s medium (DMEM; Sigma-Aldrich, USA) supplemented with 10% fetal bovine serum (FBS) (Gibco, Thermo Fischer Scientific, Waltham, MA, USA), 100 U/mL penicillin, and 100 μg/mL streptomycin at 37 °C in a 5% CO_2_ atmosphere (Panasonic MCO-19AIC incubator, Osaka, Japan). Cells were used for the experiments during 4–8 passages after thawing. For the experiments, cells were washed twice with a calcium- and magnesium-free buffered saline solution (CMF-BSS), detached from the flasks using trypsin/EDTA (Gibco, Thermo Fischer Scientific, USA), and suspended in the supplemented medium. For the viability assay, cells (5 × 10^4^ cells/cm^2^) were added to 96-well microplates (0.2 mL final volume) and incubated with different concentrations of *Ro* for 24 h. After adding 0.25 mg/mL MTT [3-(4,5-dimethylthiazol-2-yl)-2,5-diphenyl-2H-tetrazolium bromide] (Sigma-Aldrich, USA), 4 h of incubation, and the solubilization of formazan crystals, the absorbance of each well was measured at 570 nm in a Biotek ELX 800 microplate reader (BioTek Instruments, Winooski, VT, USA). Cell viability was determined relative to the control (absence of *Ro*), which was considered to have a 100% viability [58]. The mitochondrial transmembrane potential in the BRL 3A cells was estimated using fluorescence microscopy. Cells (5 × 10^4^ cells/cm^2^) were seeded in 3.5 cm glass dishes (with a 0.17 mm thick cover glass on the bottom (Greiner Bio-One, Frickenhausen, Germany) and loaded with 40 nM DiOC_6_(3) (Thermo Fisher Scientific, USA) and 5 nM Hoechst 33258 (Thermo Fisher Scientific, USA) with incubation at 37 °C for 30 min. Images were acquired in a widefield Leica AF6000 microscope (Leica Microsystems, Wetzlar, Germany), using the HCX APO UVI 100×/1.3 oil plan apochromatic objective and the ultrafast Leica DFC365 FX monochromatic digital camera (Leica Microsystems, Germany). The set of cube filters used included A4 (ex 360/40, DC 400 nm, em 470/40) and L5 (ex 480/40, DC 505, em 527/30) (Leica Microsystems, Germany) filters.

### 2.10. Blood Biochemical Analyses

Aspartate aminotransferase (AST) and alanine aminotransferase (ALT) were determined in serum using commercial kits according to the manufacturer’s instructions (LABTEST, Lagoa Santa, MG, Brazil).

### 2.11. Untargeted Metabolomics Analysis by HPLC-ESI-MS/MS

An HPLC-MS analysis was performed in a Shimadzu UFLC system coupled to a quadrupole time-of-flight mass spectrometer (micrOTOF QII, Bruker Daltonics, Billerica, MA, USA) using a C18 column (5 µm, XB-C18 Kinetex, 100Å, 150 × 3 mm, Phenomenex). The mobile phase was composed of water (A) and MeOH (B), both with 0.1% formic acid, at a flow rate of 0.75 mL.min^−1^. The following gradient was employed: 0–23 min, 10–100% B; 23–26 min, 100% B; 26–27 min, 100–10% B; and 27–30 min, 10% B. The column oven was set to 45 °C, and an injection volume of 10 µL was selected. Chromatograms were acquired in both positive and negative ionization modes, and the following parameters were employed for the mass spectrometer: end plate offset, 500 V; capillary voltage, 3200 V for negative mode and 3500 V for positive mode; nebulizer pressure, 4.5 bar; dry gas (N_2_) flow, 9 L.min^−1^; dry temperature, 200 °C; mass range, *m*/*z* 150 to 1200; MS/MS scan mode; number of precursors, 3; exclusion activation, 1 spectrum; and exclusion release, 36 s.

### 2.12. Gas Chromatography-Mass Spectrometry (GC-MS) Analysis

The glycolic extract was submitted to a head space analysis, and the GC-MS methodology was based on the previously reported methodology [59]. The analysis was carried out on the GCMS-QP2010 (Schimadzu, Japan) according to the following parameters: injector temperature, 250 °C; column temperature, 60 °C; heating ramp from 60 to 210 °C, at 3 °C/min, with a total time of 50 min; chromatographic column, DB-5, 30 m × 0.25 mm in diameter, 0.25 μm in thickness; and helium was used as the carrier gas under 79.7 kPa at 1.30 mL/min with a linear velocity of 41.6 cm/s, a 1 μL injection volume, and a 1:60 split.

### 2.13. Statistical Analyses

Quantitative data are presented as the mean ± SD of (at least) three independent experiments performed in triplicate. For multiple comparisons, a one-way ANOVA was used, followed by Tukey’s post hoc test. The Prism 8.0 software (GraphPad Software Inc., La Jolla, CA, USA) was used to perform the data analyses. Statistical significance was defined as * (*p* < 0.05), ** (*p* < 0.01), and *** (*p* < 0.001).

## 3. Results

### 3.1. Rosmarinus officinalis Glycolic Extract (Ro) Protects Mitochondrial Lipids and Proteins from Oxidation

**Figure 1:**
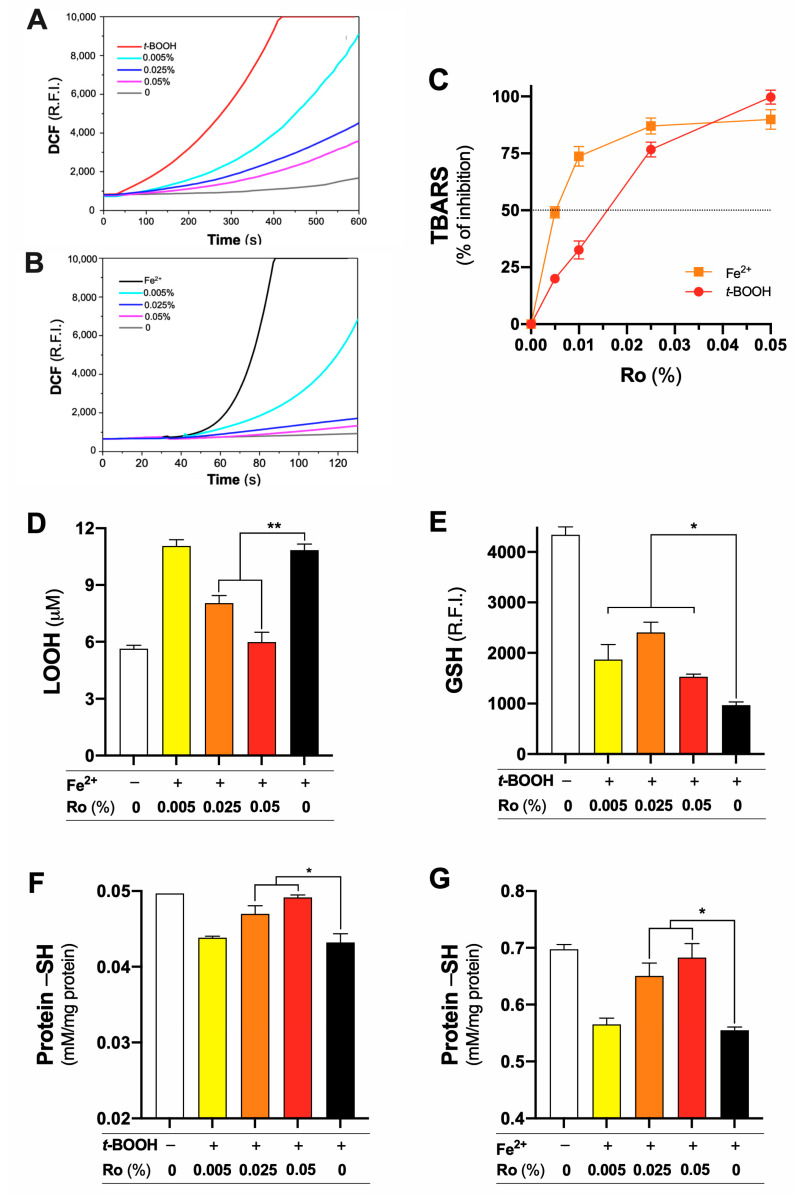
Glycolic extract of *Rosmarinus officinalis* (*Ro*) protects isolated rat liver mitochondria from oxidative stress. Mitochondria (1.0 mg/mL) were energized by succinate (site II substrate) in the presence of rotenone. DCF fluorescence was recorded kinetically using 0.6 mM *t*-BOOH ((**A**), red line) or 50 mM Fe^2+^ ((**B**), black line) as inducers of oxidative stress. *Ro* concentrations are represented by cyan (0.005%), blue (0.025%), and magenta (0.05%) lines. The inhibitory effect of *Ro* on the lipid oxidation (TBARS) induced by Fe^2+^ (squares, orange line) or *t*-BOOH (circles, red line). Effects of different concentrations of *Ro* on the formation of lipid hydroperoxides (LOOH) induced by Fe^2+^ (**D**), GSH oxidation induced by *t*-BOI (**E**), and oxidation of protein thiol groups induced by *t*-BOOH (**F**) or Fe^2+^ (**G**). In (**D**–**G**), negative control (absence of Fe^2+^ and *Ro*) is represented by white bars, positive control (Fe^2+^ or *t*-BOOH) by black bars, and the effects of *Ro* by yellow (0.005%), orange (0.025%), and red (0.05%) bars. In (**C**–**G**), data are presented as mean ± SD of three independent experiments. Statistical differences are indicated by * (*p* < 0.05) and ** (*p* < 0.01).

The production of mitochondrial ROS was evaluated through the kinetic measurement of dichlorofluorescein (DCF) fluorescence. When incubated with *t*-BOOH or Fe^2+^, isolated rat liver mitochondria energized by succinate immediately increased the production of ROS. Due to the nature of the oxidative stress inducer, it was possible to observe that the fluorescence increase rate was faster for the Fe^2+^ (Figure 1B, black line) than for the *t*-BOOH (Figure 1A, red line). The preincubation of the mitochondrial suspension with *Ro* abolished the ROS production triggered by both inducers in a concentration-dependent manner. These effects were translated into the protection of mitochondrial membranes from oxidation by Fe^2+^ (squares, orange) or *t*-BOOH (circles, red), estimated by TBARS (Figure 1C). Moreover, the formation of Fe^2+^-induced lipid hydroperoxides was inhibited by *Ro* at 0.025% (orange) and 0.05% (red) (Figure 1D). Such a lipid protective action exhibited by *Ro* was accompanied by the prevention of GSH oxidation by *t*-BOOH (Figure 1E) and the oxidation of reduced thiol groups of mitochondrial proteins by both *t*-BOOH (Figure 1F) and Fe^2+^ (Figure 1G) at the same concentrations.

Together, these results indicate the ability of *Ro* to protect mitochondria (and possibly cells) from oxidative damage, highlighting its potential to prevent pathological conditions and diseases associated with oxidative stress.

### 3.2. Iron (II) Chelating and Free Radical Scavenger Activity Account to the Antioxidant Protection Exhibited by Ro

**Figure 2:**
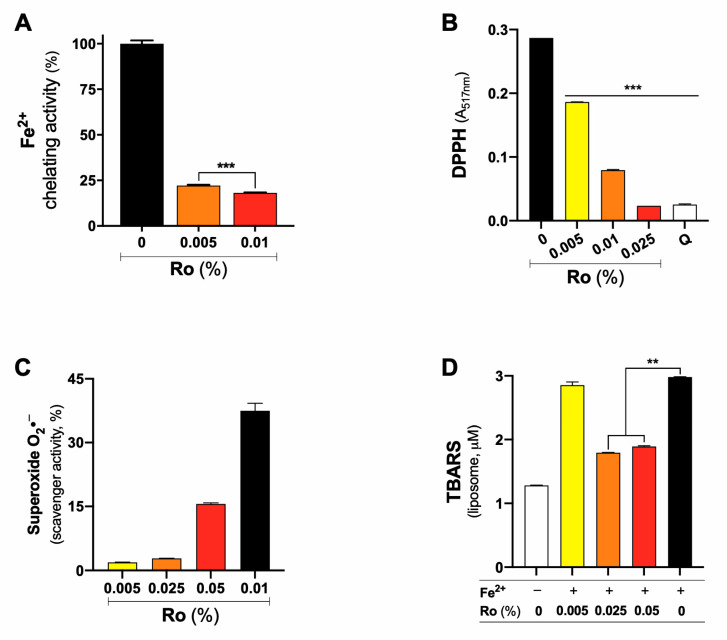
Fe^2+^ chelating and free radical scavenger activities of glycolic extract of *Rosmarinus officinalis* (*Ro*). (**A**) 0.005 or 0.01% *Ro* were incubated with 50 μM Fe(NH_4_)_2_(SO_4_)_2_ and the absorbance of bathophenanthroline-Fe^2+^ complex was determined at 535 nm. (**B**) DPPH reduction by different *Ro* concentrations and the absorbance were determined at 517 nm, using 10 μM quercetin (Q) as a reference. (**C**) Superoxide was detected by its reaction with nitroblue tetrazolium (NBT) at 540 nm, considered 100% in the absence of *Ro*; different indicated *Ro* concentrations decreased NBT reduction. (**D**) Inhibitory effect of *Ro* on the lipid oxidation (TBARS) of 1.0 mM PCPECL liposomes induced by Fe^2+^. Results expressed as percentage were calculated in relation to control (absence of *Ro* or Fe^2+^), considered to be 100%. Data are presented as mean ± SD of three independent experiments. Statistical differences are indicated by ** (*p* < 0.01) and *** (*p* < 0.001).

The quantification of total phenols and flavonoids, substances with recognized antioxidant action, are presented in Table 1. In an attempt to provide further mechanistical insights regarding the antioxidant capacity of *Ro*, its ability to chelate Fe^2+^, which is used here as inducer of oxidative stress, was investigated. In a competitive spectrophotometric assay using bathophenanthroline, *Ro* chelated more than 75% of the available Fe^2+^, even at a lower concentration (Figure 2A). This may help to explain, at least partially, the protective effects observed in the Figure 1. Additionally, the free radical scavenger activity of *Ro* was investigated. As observed in Figure 2B, *Ro* reduced (scavenged) DPPH radicals in a concentration-dependent manner. At 0.025%, the effect was similar to 10 μM quercetin, a well-studied flavonoid known for its free radical scavenger activity and antioxidant properties [60]. Since DPPH is not a biological free radical, we also investigated the ability of *Ro* to scavenge O_2_^•−^ generated by the xanthine/xanthine oxidase system, using NBT as indicator [61]. *Ro* exhibited significant O_2_^•−^ scavenger activity at 0.05 and 0.01%, evaluated by the inhibition of NBT reduction by this radical. Finally, using a lipidic model system to mimic mitochondrial membranes [62], i.e., unsaturated PCPECL liposomes, it was possible to demonstrate that *Ro* prevents Fe^2+^-induced lipid oxidation regardless of mitochondrial function or a dependence on mitochondrial constituents. Thus, the ability to scavenge free radicals and to block lipid peroxidation reactions contributed to *Ro*’s antioxidant activity and to the protective effects observed in mitochondria (Figure 1).

### 3.3. Ro Does Not Affect Viability or Mitochondrial Membrane Potential (∆Ψ) of Liver Fibroblasts

**Figure 3:**
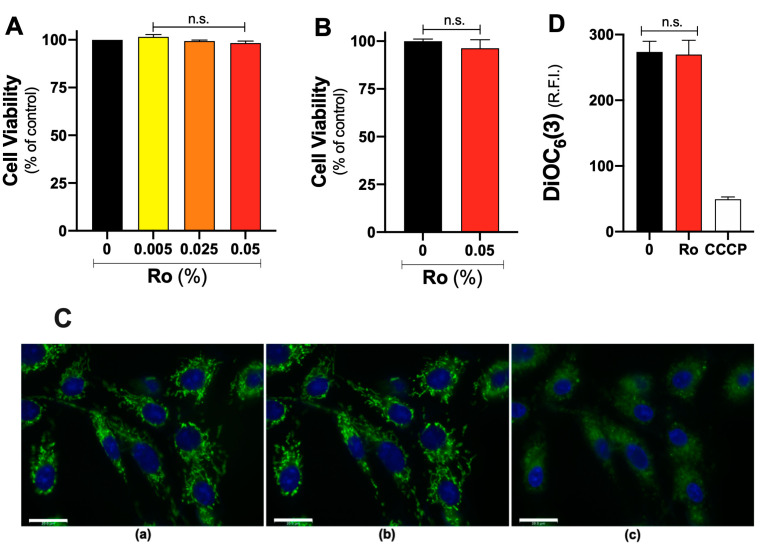
Absence of cytotoxicity of glycolic extract of *Rosmarinus officinalis* (*Ro*). (**A**) Viability of BRL 3A cells incubated with *Ro* (0 to 0.05%) for 24 h was estimated with MTT (**A**) and trypan blue (**B**) assays. The effects of *Ro* on cell viability were calculated in relation to control (absence of *Ro*), considered 100%. (**C**) Representative images of the mitochondrial membrane potential estimated by fluorescence microscopy using DiOC_6_(3). Cells were loaded with dyes, and fluorescence emission was acquired before and after 0.05% *Ro* addition. The uncoupler CCCP (10 μM) was used as positive control (see Methods for details). Scale bars (20 μm) (**D**) Relative quantification of DiOC_6_(3) fluorescence emission (L5 channel) considering the replicates. In (**A**,**B**) data are expressed as percentages of viable cells (mean ± SD) calculated in relation to control (i.e., without *Ro*), considered as 100%. Statistically non-significant (n.s.), considering *p* < 0.05.

To strengthen the pharmacological potential of *Ro*, its cytotoxicity was evaluated in vitro. This is relevant since many antioxidant substances also have a cytotoxic effect [63] which can hinder their therapeutic potential. In this regard, the possible cytotoxicity of *Ro* was investigated in cultured BRL 3A cells, a fibroblast-like cell line isolated from rat livers. Cell viability, evaluated by the MTT assay, was not affected by *Ro* in the concentration range tested (0.005–0.05%), i.e., the incubation of *Ro* with BRL3A cells for 24 h did not decrease cell viability compared to the control (Figure 3A). These results were confirmed by the Trypan blue exclusion assay (Figure 3B). Moreover, *Ro* did not alter the ∆Ψ of BRL 3A cells assessed by fluorescence microscopy using DiOC_6_(3) (Figure 3C(b),D, red bar). As a control, the total ∆Ψ dissipation was achieved by adding CCCP, an uncoupler of the OXPHOS (Figure 3C(c),D, white bar).

### 3.4. Ro Protection against Acetaminophen-Induced Hepatotoxicity in Rats

**Figure 4:**
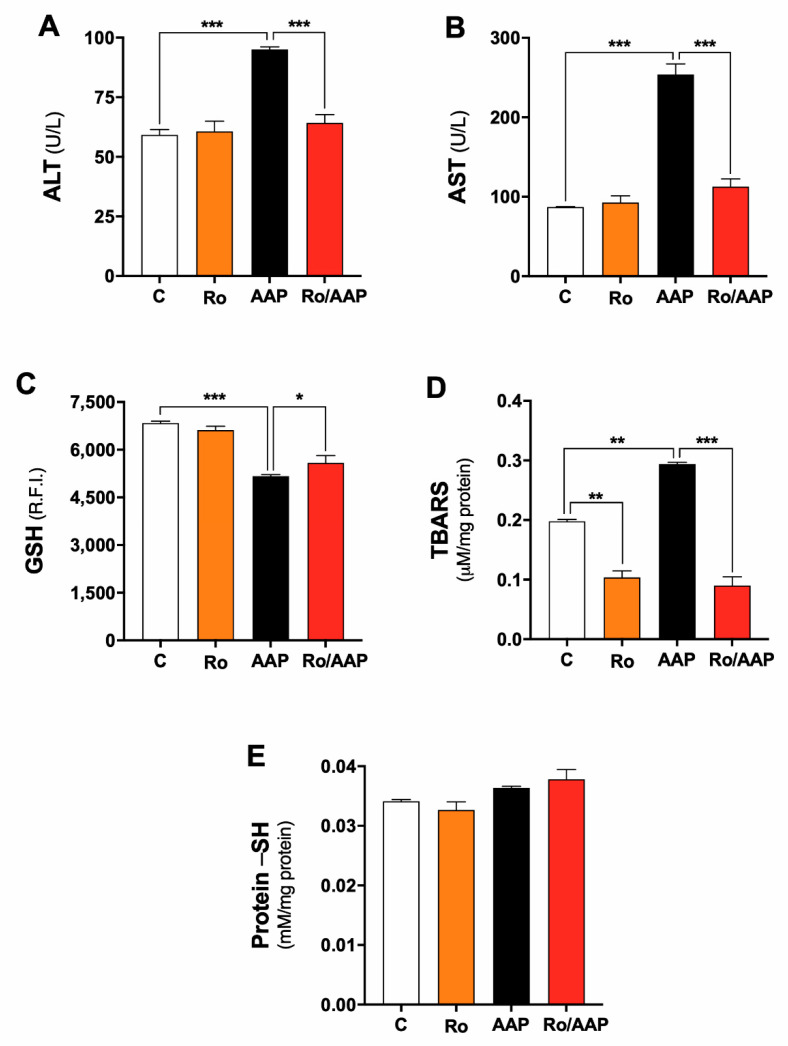
*Rosmarinus officinalis* (*Ro*) protects against acetaminophen-induced hepatotoxicity in vivo. Rats were divided into four groups (n = 7) and treated intraperitoneally, as described in Materials and Methods. Group C (white bars): control, treated with vehicle (DMSO); group *Ro* (orange bars): 0.5% *Ro* once a day for four days; group AAP (black bars): single dose of 900 mg/kg acetaminophen; group Ro/AAP (red bars): received *Ro* for four days, followed by single dose of AAP. (**A**) Alanine aminotransferase and (**B**) aspartate aminotransferase levels. (**C**) GSH, (**D**) TBARS, and (**E**) reduced thiol groups of proteins quantified as described by isolated mitochondria in Figure 1. Statistical differences are indicated by * (*p* < 0.05), ** (*p* < 0.01), and *** (*p* < 0.001).

The antioxidant activity of *Ro*, its effect in protecting isolated rat liver mitochondria from oxidative stress conditions, and its absence of cytotoxicity in vitro led us to investigate whether such protective effects can be observed in vivo. To this end, a well-established and clinically relevant model of oxidative-stress-mediated hepatotoxicity was selected. The acute exposure to high doses of AAP generates radical intermediates during its hepatic metabolism, depleting antioxidant defenses and resulting in oxidative tissue damage [43]. To establish the experimental model, a single dose of AAP (900 mg/kg) was administrated to the rats, which were or were not treated with 0.5% *Ro*, and the blood levels of alanine aminotransferase (ALT) and aspartate aminotransferase (AST) were analyzed. This high dose of AAP is well established in the literature to induce acute hepatotoxicity in mice and rats [64,65]. As expected, AAP administration increased serum levels of ALT (Figure 4A, black bar) and AST (Figure 4B, black bar), indicating the occurrence of liver injury. As a control, *Ro* alone did not exert any effect on ALT or AST levels (orange bars, Figure 4A,B, respectively) compared to the basal levels, i.e., control, non-treated rats (white bars, Figure 4A,B), indicating the absence of an intrinsic hepatotoxicity of the extract.

Such a hepatotoxic effect of AAP was accompanied by GSH consumption (Figure 4C, black bar) and increased lipid oxidation (Figure 4D, black bar) without affecting the reduced thiol levels of proteins (Figure 4E, black bar). Interestingly, the treatment of animals with 0.5% *Ro* was able to reestablish the basal levels of ALT and AST in AAP-treated animals (red bars, Figure 4A,B, respectively), indicating a complete restoration of the liver integrity against the AAP-induced toxicity. We then further investigated whether this effect was accompanied by an improvement in oxidative stress indicators. In AAP-treated rats, *Ro* partially inhibited GSH oxidation (Figure 4C, red bar) and completely prevented lipid oxidation (Figure 4D, red bar) without an effect on the redox state of the protein thiol groups (Figure 4E, red bar). It is noteworthy that *Ro* diminished even the basal lipid oxidation (Figure 4D, orange bar).

### 3.5. Chemical Composition of Rosmarinus officinalis L. Glycolic Extract

**Figure 5:**
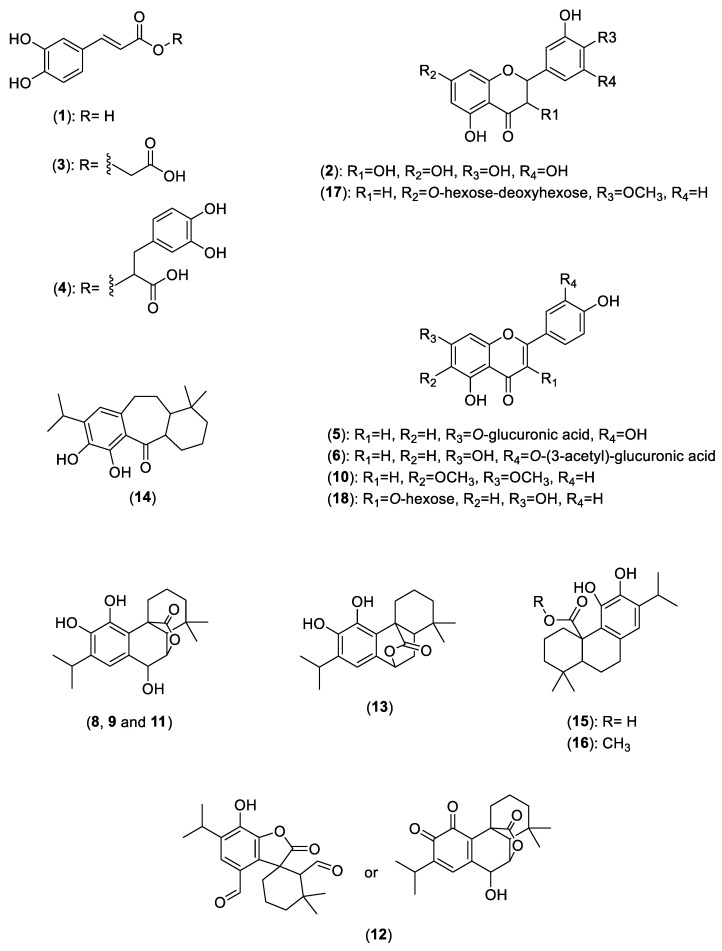
Chemical structures of compounds annotated in the *Ro* extract. Numbers in each structure refer to those presented in Table 2.

**Figure 6:**
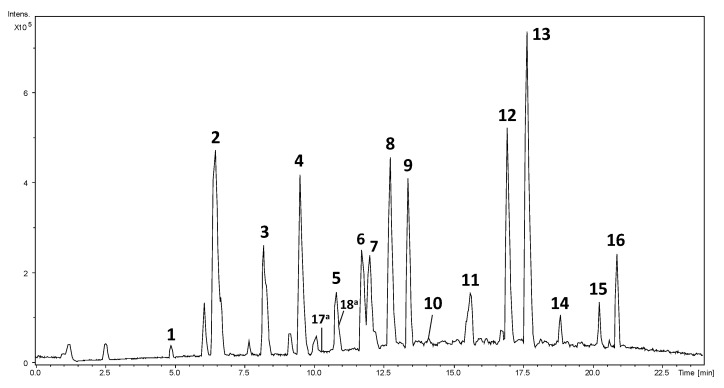
Total Ion Chromatogram (TIC) of Ro obtained by LC-ESI-MS/MS analysis (negative mode). ^a^ Annotations from LC-ESI-MS/MS analysis in positive mode.

In order to gain more information about the chemical composition of *Ro*, we conducted an untargeted metabolomics analysis of the glycolic extract at both positive and negative ionization modes. The annotated metabolites are shown in Figure 5/Table 2 and the ion intensity of each metabolite are presented in Figure 6. As expected, several phenolic compounds already described for this plant were detected [36], e.g., caffeic acid, rosmanol, and rosmarinic and carnosinic acids. All metabolites had their annotations confirmed through a detailed discussion of the decomposition reactions in the gaseous phase [66], following the correlation with the literature recently proposed by Pilon and collaborators [67] for the analysis of glycosylated flavonoids.

## 4. Discussion

The wide range of biological actions reported for *R. officinalis* has long drawn the attention of researchers worldwide. In this study, for the first time, we showed that the antioxidant activity exhibited by rosemary extract and its chemical constituents is able to protect mitochondria subject to oxidative stress conditions. During the electron transport by the respiratory chain, mitochondria generate superoxide anions that are converted to hydrogen peroxide (H_2_O_2_). In the presence of Fe^2+^, H_2_O_2_ is converted to hydroxyl radicals, and these species are able to oxidize mitochondrial lipids and proteins, compromising energy production and Ca^2+^ homeostasis and culminating in cell death [46,72]. Thus, we used this complex system to evaluate the antioxidant potential of the glycolic extract of *R. officinalis* (*Ro*). Two different methods of inducing oxidative stress were used: Fe^2+^ and *t*-butyl hydroperoxide (*t*-BOOH). The former is able to catalyze Fenton reactions, amplifying the production of reactive oxygen species (ROS). and the latter is an organic peroxide that per se consumes antioxidant defenses to be eliminated, exposing cells to oxidative damage [45,46]. In both situations, *Ro* was able to inhibit ROS production and the oxidation of mitochondrial lipids and proteins. Previous studies have reported the ability of *R. officinalis* to inhibit the lipid peroxidation of phospholipids and membrane model systems [73,74,75], erythrocytes [76,77], low density lipoproteins (LDL) [78], and tissue homogenates [28].

Among the mechanisms of action for antioxidant activity, we found that *Ro* exhibits a free radical scavenger activity and the ability to chelate Fe^2+^. Most studies have used a DPPH assay to infer the scavenger activity of rosemary extracts and essential oils; however, it is noteworthy that DPPH is not a radical produced in biological systems. Therefore, we further examined and demonstrated the capacity of the rosemary leaf glycolic extract to scavenge superoxide anions. Such an ability was previously shown for diterpenoids isolated from *R. officinalis* [79], and it is subject to seasonal variations [80]. The essential oil of *R. officinalis* was also able to scavenge hydroxyl radicals and chelate Fe^2+^ [81]. These properties of *R. officinalis* extracts are provided by their high content of powerful, well-known, synergistically acting antioxidant compounds including flavonoids, phenolic acids, and terpenes. For example, the antioxidant activity exhibited by carnosol was linked to an enhanced health and lifespan in *C. elegans*, accompanied by an increase in the activity of several antioxidant enzymes and a decrease in TBARS content [82]. As expected, the phytochemical analysis of the glycolic extract of *R. officinalis* identified the presence of several phenolic compounds already described for this plant. It should be noted that chlorogenic acid, a common compound with significant antioxidant activity, was not observed in these samples. A recent study demonstrated that the concentration of chlorogenic acid varies greatly depending on the drying process used for the leaves [83], which may explain, in part, the absence of the signal. The polyphenolic profile of rosemary has been widely described in the scientific literature [70,84,85,86], and the profile observed in the glycolic extract of the *R. officinalis* employed in this study was characterized by the presence of carnosic acid, carnosol, rosmarinic acid, and hesperidin. The mechanism of action was related to the free radicals’ chain terminators and scavengers of ROS. On the other hand, compounds such as hesperidin are known for their ability to chelate metals. This confers a resistance to pests, as described in citrus [87], but can also support antioxidant properties. The occurrence of astragalin reinforces all the biological effects described in our article. This kaempferol 3-glucoside has several biological activities described, including antioxidant effects [88]. Therefore, the global analysis of the main metabolites contained in the extract support the observed activities. Finally, as expected, GC-MS exhibited a trace signal. The major compound detected was camphor, showing no contribution of the previous essential oil constituents to the described biological activities.

The antioxidant power of *R. officinalis* has been shown to be responsible for the plant’s protective effects against oxidative stress. Rosemary prevented the renal toxicity elicited by diethylnitrosamine [89] and potassium dichromate [90] by inhibiting lipid peroxidation and improving the capacity of the antioxidant enzymatic system. Potassium dichromate was also employed to induce hepatotoxicity in rats. In this context, rosemary essential oil exhibited hepatoprotective action, characterized by the inhibition of the lipid peroxidation, GSH, and protein oxidation, and by the restoration of the levels of antioxidant enzymes [40]. Similar observations were made for hepatotoxicity induced by CCl_4_. Rosemary essential oil inhibited the lipid peroxidation and ‘reversed’ the activities of antioxidant enzymes catalase, peroxidase, glutathione peroxidase, and glutathione reductase in a liver homogenate [91]. A methanolic extract obtained from *R. officinalis* leaves [92,93] and a shoot tincture [94] also presented the same protective results on CCl_4_-induced hepatotoxicity in rats. When *t*-BOOH was used as the oxidizing agent used to induce liver damage, *R. officinalis* also exhibited protective effects [95]. Acetaminophen is one of the most widely used analgesic and antipyretic drugs in the world. Although it is a relatively safe drug at therapeutic doses, acute overdose, chronical exposition, or association with other xenobiotics can result in severe hepatotoxicity. After oral intake, a major part of it is conjugated with glucuronic acid in the liver and eliminated by the kidneys. However, a fraction of AAP undergoes metabolization by the cytochrome P_450_ system, generating the toxic metabolite N-acetyl-p-benzoquinone imine (NAPQI) which, under normal conditions, is conjugated with GSH and eliminated. Nonetheless, increased NAPQI production results in the depletion of GSH and, consequently, oxidative liver damage [96]. Thus, we investigated for the first time the possible protective effect of *Ro* on AAP-induced hepatotoxicity. To validate the model, our data showed that a single high dose of AAP caused a depletion of GSH and increased lipid peroxidation. Such oxidative alterations were accompanied with liver damage. This was attested by increased levels of ALT and AST, biochemical markers of hepatocyte damage. Both aminotransferases were quantified since tissues other than the liver have elevated AST levels, such as the erythrocytes, heart, and muscle, while ALT has more specificity to the liver. Our data showed an increased AST/ALT ratio (De Ritis ratio), higher than 1.0, when rats were treated with AAP. This is predictive of long-term complications such as fibrosis and cirrhosis [97]. As shown, *Ro* indisputably protected the rat livers from AAP toxicity since the AST and ALT levels returned to the basal levels when compared to control. The powerful antioxidant activity of *Ro* seems to be responsible for this hepatoprotection since the GSH depletion was abolished and the lipid oxidation was also diminished. This strategy of using antioxidants to protect the liver from AAP toxicity was successfully achieved with N-acetyl cysteine [98,99].

Finally, it is important to correlate the general chemistry data annotated in this paper with the *Ro* effects. More than half of the compounds have the catechol group. It is well known that catechol and its many functionalized derivatives are excellent metal chelators [100]. Considering only the phenol group, which is a good and well-known antioxidant [100], the number is even more expressive since all 18 major compounds noted have at least one hydroxyl linked to an aromatic ring. Therefore, we believe that the observed effect must surely be a synergistic combination of all the actives described in this work.

## 5. Conclusions

Together, our data reinforced the antioxidant activity of *R. officinalis* through a free radical scavenger activity and by diminishing the Fe^2+^-catalyzed Fenton reactions. We showed that these activities enabled *Ro* to protect isolated rat liver mitochondria from oxidative damage caused by different mechanisms (Fe^2+^ or *t*-BOOH). Moreover, *Ro* did not present cytotoxicity in cultured liver cells, and it exhibited a potent hepatoprotective effect against AAP toxicity in vivo. Further studies are necessary to the development of pharmaceutical formulations containing Ro, and clinical trials are required to evaluate the potential hepatoprotective effect in patients.

## Figures and Tables

**Table 1:** Total phenols and flavonoids concentration present in 0.05% *Ro*.

	Concentration (μM)
Total phenols	12.4 ± 0.2
Flavonoids	7.2 ± 0.1

**Table 2:** Metabolites of *Ro* annotated by HPLC-ESI-MS/MS.

ID	RT (min)	MS *m/z* [M − H]^−^	MS/MS *m/z*	Compound	Reference
**1**	4.9	179	135	Caffeic acid	[68]
**2** ^a^	6.5	305	225	Gallocatechin	[69]
**3**	8.2	237	179, 161	Caffeoylglycolic acid	-
**4** ^a^	9.4	359	161(100), 179, 197	Rosmarinic acid	[70]
**5**	10.8	461	285(100), 199, 151	Luteolin-7-*O*-glucuronide	[68]
**6**	11.7	503	399, 285(100), 255	Luteolin-3′-*O*-(3-acetyl)-glucuronide	[11]
**7**	12.0	315	300(100), 227, 199	Methoxy-tetra-hydroxy-flavone	-
**8** ^a^	12.8	345	301, 283, 267, 217	Rosmanol isomer	[70]
**9** ^a^	13.4	345	283, 267, 227	Rosmanol isomer/epirosmanol	[70]
**10**	14.1	313	298, 283, 255, 227, 164	Cirsimaritin	[71] (CCMSLIB00004718157)
**11**	15.6	345	283, 227	Rosmanol isomer/epirosmanol	[70]
**12** ^a^	16.9	343	299, 243, 216	Rosmadial or Rosmanol quinone	[68]
**13** ^a^	17.7	329	285(100), 201	Carnosol	[70]
**14**	18.9	315	285, 201	Rosmaridiphenol	[68]
**15**	20.3	331	287(100), 272, 244	Carnosic acid	[68]
**16**	20.9	345	301(100), 286, 271	Methyl carnosate	[70]
	RT (min)	MS *m/z* [M + H]^+^	MS/MS *m/z*	Compound	Reference
**17**	10.3	611	547, 449, 287(100)	Hesperidin	[71] (CCMSLIB00000214340)
**18**	10.8	449	287(100)	Astragalin	[71] (CCMSLIB00003136634)

**^a^** Majority compounds—TIC (negative mode).

## Data Availability

Not applicable.
